# Supplementary material for: Clinical profiling of specific diagnostic subgroups of women with chronic pelvic pain
Source: Front Reprod Health. 2023 May 30;5:1140857. doi: 10.3389/frph.2023.1140857 (PMC10266100; doi:10.3389/frph.2023.1140857)
Supplement: Supplementary file 6 [file Table6.pdf]

	EAP		EABP		BPS		PP	
	N	%	N	%	N	%	N	%
**Pain interference with:**								
Work or school								
Low	19	16.1	12	13.8	7	17.5	13	31.7
Medium	16	13.6	20	23	8	20	4	9.8
High	52	44.1	46	52.9	13	32.5	8	19.5
Daily activities at home								
Low	26	22.2	12	13.6	9	22.5	10	24.4
Medium	23	19.7	22	25	9	22.5	8	19.5
High	47	40.2	50	56.8	14	35	8	19.5
Sleep								
Low	29	25.4	15	16.7	9	22.5	11	27.5
Medium	24	21.1	17	18.9	6	15	7	17.5
High	36	31.6	55	61.1	18	45	7	17.5
Sexual Intercourse								
Low	15	12.9	4	4.7	5	12.8	6	14.6
Medium	12	10.3	5	5.8	5	12.8	6	14.6
High	38	32.8	52	60.5	18	46.2	7	17.1
Exercise/sports								
Low	23	19.5	9	10.1	10	25.6	16	39
Medium	25	21.2	17	19.1	2	5.1	5	12.2
High	52	44.1	50	56.2	19	48.7	7	17.1
Social activities								
Low	31	26.7	15	17.4	10	25.6	14	34.1
Medium	20	17.2	16	18.6	9	23.1	6	14.6
High	42	36.2	49	57	13	33.3	7	17.1
